# A Novel Derivative of Thioridazine Shows Low Toxicity and Efficient Activity against Gram-Positive Pathogens

**DOI:** 10.3390/antibiotics9060327

**Published:** 2020-06-15

**Authors:** Nadia S. Jørgensen, Lasse Saaby, Anne M. Andersson, Sofie Kromann, Ehsan Sheikhsamani, Anders Permin, Troels Ronco, Søren W. Svenningsen, Jørn B. Christensen, Rikke H. Olsen

**Affiliations:** 1 Department of Veterinary and Animal Sciences, University of Copenhagen, Stigboejlen 4, 1870 Frederiksberg C, Denmark; dst742@alumni.ku.dk (N.S.J.); annemarlenealexandra@gmail.com (A.M.A.); sok@sund.ku.dk (S.K.); troelsronco@gmail.com (T.R.); 2 Bioneer:FARMA, Department of Pharmacy, Universitetsparken 2, 2100 Copenhagen, Denmark; lasse.saaby@sund.ku.dk; 3 Department of animal science, Ferdowsi University, Mashhad IR-91779, Iran; e_sh_s@yahoo.com; 4 Unibrains, Kollelevbakken 33, 2830 Virum, Denmark; aper@unibrains.dk; 5 Department of Chemistry, University of Copenhagen, Thorvaldsensvej 40, 1871 Frederiksberg C, Denmark; swsvenningsen@hotmail.com (S.W.S.); jbc@chem.ku.dk (J.B.C.)

**Keywords:** thioridazine, novel antimicrobial compound, Gram-positive pathogens

## Abstract

Thioridazine hydrochloride (HCl) has been suggested as a promising antimicrobial helper compound for the treatment of infections with antimicrobial-resistant bacteria. Unfortunately, the therapeutic concentration of thioridazine HCl is generally higher than what can be tolerated clinically, in part due to its toxic side effects on the central nervous system. Therefore, we aimed to synthesize a less toxic thioridazine derivative that would still retain its properties as a helper compound. This resulted in a compound designated 1-methyl-2-(2-(2-(methylthio)-10H-phenothiazin-10-yl)ethyl)-1-pentylpiperidin-1-ium bromide (abbreviated T5), which exhibited low blood–brain barrier permeability. The lowest minimal inhibitory concentration (MIC) against *Staphylococcus aureus* exposed to the novel compound was reduced 32-fold compared to thioridazine HCl (from 32 µg/mL to 1 µg/mL). The MIC values for T5 against five Gram-positive pathogens ranged from 1 µg/mL to 8 µg/mL. In contrast to thioridazine HCl, T5 does not act synergistically with oxacillin. In silico predictive structure analysis of T5 suggests that an acceptably low toxicity and lack of induced cytotoxicity was demonstrated by a lactate dehydrogenase assay. Conclusively, T5 is suggested as a novel antimicrobial agent against Gram-positive bacteria. However, future pharmacokinetic and pharmacodynamic studies are needed to clarify the clinical potential of this novel discovery.

## 1. Introduction

Antibiotic-resistant bacteria are emerging and constitute a considerable public health issue worldwide. Since important Gram-positive and Gram-negative human and veterinary pathogens increasingly become susceptible to a wide range of antibiotics, the clinical need for new treatment compounds is urgent [1,2]. Regrettably, the pipeline for novel antimicrobials seems to have almost dried out, and the number of novel antimicrobial agents available on the market has decreased considerably in the last decades [2,3,4]. For the same reason, attention has been given to compounds that could “re-sensitize” antibiotic resistant bacteria to the currently available antibiotics [5,6,7]. Phenothiazines, a group of neuroactive compounds, have been intensively studied as antimicrobial helper compounds, and have been documented to re-sensitize methicillin-resistant *Staphylococcus aureus* (MRSA) to oxacillin in vitro [8,9]. Unfortunately, the promising results produced in vitro have proven difficult to reproduce in vivo [10,11]. This is most likely due to the toxic side effects of phenothiazines in the needed concentrations to obtain synergy. At least one study has reported that the animal experiment had to be terminated prematurely, due to severe behavioral side effects in the phenothiazine-treated animals [10,11]. We have hypothesized that part of the toxicity of phenothiazines is due to the compounds adverse effects on the brain, as they readily pass the blood–brain barrier (BBB) [12]. Therefore, the aim of this study was to chemically modify thioridazine hydrochloride (HCl) to generate a thioridazine derivative that would not pass the BBB, but retain its properties as an antimicrobial helper compound.

## 2. Results

### 2.1. Structure, Yield, and Chemical Characteristics of T5

The product yield of the compound was 0.44g (51%), with the following characteristics: ^1^H NMR (500 MHz, CD_3_OD: δ 7.26 (*t*, *J* = 7.1 Hz, 1H), 7.23–7.17 (*m*, 1H), 7.15–7.06 (*m*, 2H), 7.04–6.98 (*m*, 1H), 6.97–6.94 (*m*, 1H), 6.91 (*d*, *J* = 7.3 Hz, 1H), 4.22 (*d*, *J* = 13.4 Hz, 1H), 4.10–3.95 (*m*, 1H), 3.59–3.32 (*m*, 3H), 3.26 (*td*, *J* = 12.9, 3.0 Hz, 1H), 3.06 (*tt*, *J* = 10.6, 7.5 Hz, 2H), 2.92 (*s*, 2H), 2.80 (*s*, 1H), 2.60–2.52 (*m*, 1H), 2.49 (*s*, 3H), 2.01–1.69 (*m*, 6H), 1.63–1.43 (*m*, 2H), 1.43–1.14 (*m*, 4H), 1.08–0.94 (*m,* 2H), 0.94–0.83 (*m*, 3H); and ^13^C-NMR (126 MHz, CD_3_OD) δ 146.85, 146.08, 140.45, 140.36, 129.01, 128.97, 128.78, 128.70, 127.68, 124.60, 123.95, 122.20, 117.82, 117.68, 115.64, 115.52, 68.50, 65.80, 63.05, 61.79, 50.30, 44.78, 43.91, 42.63, 29.60, 29.52, 26.37, 26.17, 25.96, 23.37, 23.15, 22.79, 22.11, 21.99, 21.04, 21.01, 15.90, 15.86, 14.29, 14.21.

**Scheme 1:**
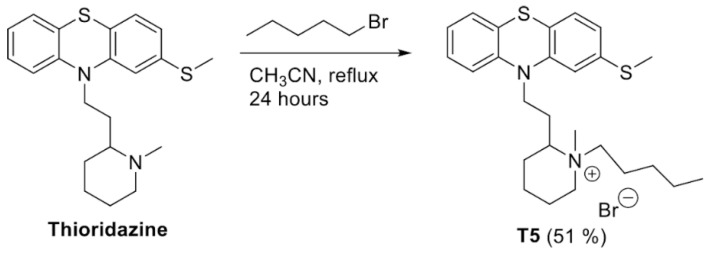
Synthesis of 1-methyl-2-(2-(2-(methylthio)-10H-phenothiazin-10-yl)ethyl)-1-pentylpiperidin-1-ium bromide (T5) from thioridazine HCl.

The found yield of HRMS (MALDI-TOF, dithranol, methanol) [C_26_H_37_N_2_S_2_] ^+^ was 441.2386 (calculated.: 441.2393). The synthesis of T5 is shown in Scheme 1.

T5 has two stereocenters, and therefore, it is a mixture of four isomers (two diastereomers and two enantiomers), which is the reason for the many signals in the ^1^H NMR and ^13^C-NMR spectra. The two enantiomers of thioridazine (2S and 2R) have previously been prepared by classical resolution of the racemate [13] by chiral HPLC [14,15]. Since previous work on the antimicrobial effect of the enantiomers of thioridazine did not show any significant difference between the two enantiomers, it was decided to test the mixture of isomers [16].

### 2.2. Cellular Permeability and Cytotoxicity of T5

**Figure 1:**
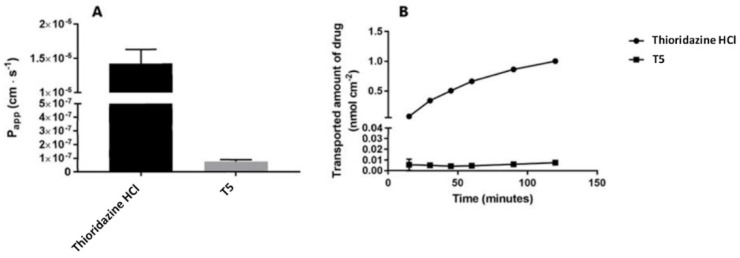
Basolateral transport assay. The figure shows apical-to-basolateral transport across IPEC-J2 MDR1 cells for thioridazine HCl and T5. (**A**) Calculated apparent permeability (P_app_); (**B**) transport across the cell monolayer over time. Values on the *y*-axis represent the amount of either thioridazine HCl or T5 appearing in the basolateral chamber at time points indicated on the *x*-axis. In both graphs, the *y*-axis has been bracketed to better visualize the results for T5.

Generally, the results show that T5 has limited cellular permeability and does not impact the monolayer integrity or induce cytotoxicity. The apical-to-basolateral transport (flux) across IPEC-J2 MDR1 cells was markedly higher for thioridazine HCl compared to T5, as shown in the permeability and flux curves (Figure 1).

The logarithmic-like shape of the flux curve for thioridazine HCl (Figure 1B) indicates that the flux of this compound was changing during the transport experiments (lack of steady-state flux). Consequently, it was difficult to calculate an accurate permeability. Permeability calculations were therefore done on smaller sections of the flux curves (at least three data points), rather than using all data points. The calculated permeabilities showed that the transport of thioridazine HCl was almost 200-fold higher than T5 (Figure 1A). Analysis of ^14^C-mannitol transport across IPEC-J2 MDR1 cells, exposed to either supplemented Hank’s balanced salt solution (HBSS; control) or 10 µM solutions of either thioridazine HCL or T5, showed that T5 did not damage the monolayer’s integrity during the transport experiment. The expected mannitol permeability across an unaffected monolayer of IPEC-J2 MDR1 cells is below 1 × 10^−6^ cm/s. Most of the values obtained in the present study were below this threshold; however, one replicate for cells exposed to supplemented HBSS and one replicate for cells exposed to 10 µM thioridazine HCl was above it. The immediate conclusion to the observed mannitol transport data would be that the barrier properties of the monolayers from which the increased mannitol transport was observed were compromised during the experiment. However, since only replicates of the two treatments showed increased mannitol transport, it seems unlikely that a possible alteration of the barrier properties should be caused by the treatment. Furthermore, all treatments were based on HBSS, and this further indicates the unlikeliness of the treatments to have had a harmful effect on the cell monolayers. It also cannot be excluded that the monolayers were compromised physically (handling) during sampling.

**Figure 2:**
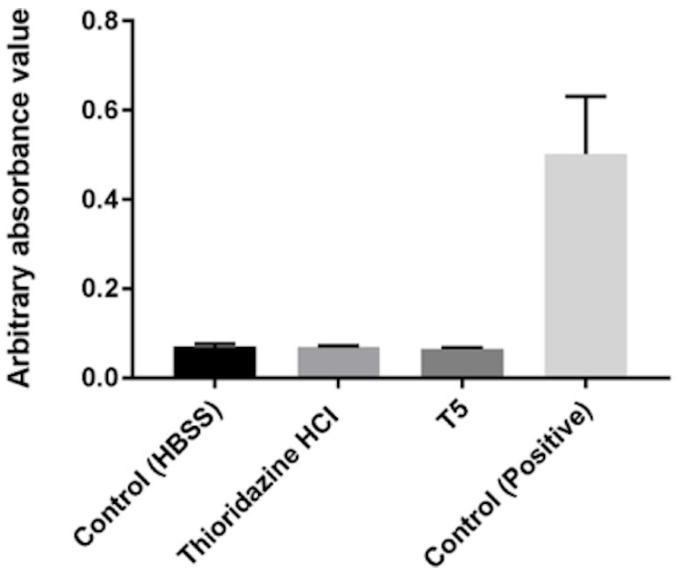
Lactate dehydrogenase (LDH)-based cytotoxicity assay. The figure shows absorbance (at 492 nm) on the *y*-axis as a measure for the lactate dehydrogenase (LDH) release from IPEC-J2 MDR1 cells exposed to thioridazine HCl and T5. Hank’s balanced salt solution (HBSS) was used as negative control, and cell lysate from IPEC-J2 MDR1 cell monolayers treated with ultrapure water was used as a positive control. The error bars indicate standard deviations of the mean values.

Lactate dehydrogenase (LDH) release was detected as a measure for cytotoxicity. In Figure 2, the absorbance data is shown as a percentage relative to the absorbance obtained from cell monolayers exposed to supplemented HBSS (negative control). Elevated LDH release was only observed for the positive control (cells treated with ultrapure water). The LDH release from IPEC-J2 MDR1 monolayers exposed to 10 µM solutions of either thioridazine HCl or T5 were comparable to HBSS. In absolute values, the absorbance measured from both the cell monolayers exposed to HBSS and the compound solutions were similar to background absorbance values (data not shown), which indicates that no detectable amounts of LDH were released from these cell monolayers. Therefore, the observations from the LDH release assay indicate that HBSS and 10 µM T5 do not cause disruption of the cell membranes.

### 2.3. Antimicrobial Activity of T5

These test results showed that T5 exhibited efficient activity against Gram-positive bacteria in the absence of human serum. The MIC value for eight different strains ranged from 1–128 µg/mL (Table 1). In all cases, the minimal bactericidal concentration (MBC) value was equal to the MIC value. Gram-negative strains (*Escherichia coli* and *Proteus vulgaris*) had MIC values at least four-fold higher than the highest MIC value observed for the Gram-positive strains. The addition of 20% serum to the assay increased the MIC and MBC at least eight-fold (Table 1).

**Figure 3:**
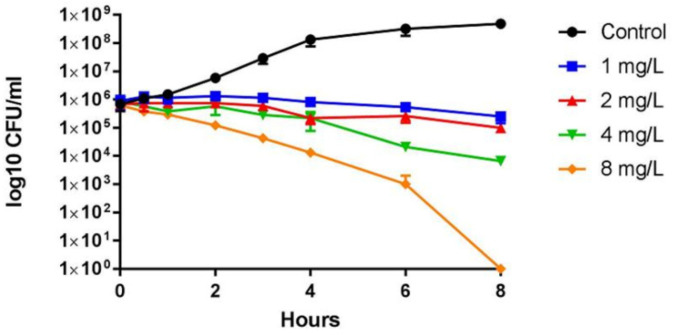
Growth and viability curves for *S. aureus* JE2. The figure shows growth and viability curves for *S. aureus* JE2 exposed to 0, 1, 2, 4, or 8 µg/mL of T5. The bacterial concentration (colony forming units (CFU)/mL) is shown on the log-transformed *y*-axis, and was determined at seven different timepoints during an eight-hour period. The experiment was repeated three times, and variations in the mean numbers of CFU/mL is indicated by the error bars.

Viability studies showed that a concentration of T5 at 8 µg/mL was sufficient to completely eliminate 10^6^ colony forming units (CFU) of *S. aureus* JE2 within eight hours, while 4 µg/mL reduced the bacterial concentration 100-fold in the same time period (Figure 3). The checkerboard assay revealed synergy between thioridazine HCl and oxacillin, which had the lowest FIC index (0.375) with the oxacillin concentration at 16 µg/mL (reduced from 128 µg/mL) and in the presence of 8 µg/mL thioridazine HCl (reduced from 32 µg/mL). The lowest FIC index when T5 was combined with oxacillin was 1; hence, there was no indication of synergy between the two compounds

### 2.4. In Silico Analysis of T5

The in silico predictions showed that T5 has a strong affinity for plasma proteins, but is not very likely to pass the BBB (Table 2). Moreover, T5 was predicted to be a non-mutagenic (Ames test) and non-carcinogenic compound that would be relatively easily absorbed intestinally and orally (Caco-2 cell permeability). However, a medium risk of blocking the human ether-a-go-go-related gene (hERG) potassium channel was found, which could indicate that T5 holds cardiotoxic properties. Compared to T5, thioridazine is more toxic regarding mutagenicity, carcinogenicity, and inhibition of hERG channels. Values regarding skin permeability, Caco-2 cell permeability, BBB penetration, and human intestinal absorption were in the same range for T5 and thioridazine. However, the data show that thioridazine has a lower affinity for plasma proteins than T5 (Table 2)

## 3. Discussion

The main motivation for synthesizing T5 was to obtain a less toxic thioridazine derivate that would not pass the BBB, yet would retain a synergistic effect with oxacillin. While we have shown that transport of T5 across monolayers of IPEC-J2 MDR1 cells was reduced by more than 100-fold compared to thioridazine HCl (Figure 1), T5 did not display synergy with oxacillin. The lack of synergy differs from the “mother” compound, which therefore could indicate a different mode of action regarding bacterial interaction. Surprisingly, the antimicrobial activity had been significantly improved for T5 compared to thioridazine HCl, at least for the Gram-positive bacterial species (Table 1). The MIC values for T5 against three MRSA strains ranged from 1–2 µg/mL (Table 1), whereas previous studies have shown that MIC values for thioridazine against MRSA strains range from 20–32 µg/mL [8,9,17]. In comparison, previous studies using a similar broth microdilution method as in this study found MIC values ranging from 0.5–1.0 µg/mL when three MRSA strains (including JE2) were tested against vancomycin, which is considered effective against MRSA [18,19,20]. Furthermore, a previous study [18] found MIC values from 0.5–2.0 µg/mL for an *Enterococcus faecium* and an *Enterococcus faecalis* strain when tested against vancomycin, whereas the MIC values for these species ranged from 4–8 µg/mL in the present study (Table 1). In contrast, the activity of T5 against Gram-negative *E. coli* strains (MIC: 32–64 µg/mL) was not remarkably improved compared to a previous study of thioridazine against *E. coli* (MIC: 128 µg/mL) [17]. In comparison, a previous study performed in accordance to CLSI’s guidelines [21] found that the majority of the *E. coli* isolates (74/85) had a MIC value ranging from 1–8 µg/mL, and EUCAST [22] has determined the MIC breakpoint for *Enterobacterales* to be 8 µg/mL in 2020, when tested against the commonly used antibiotic ampicillin. Since T5 has exhibited low activity against Gram-negative bacteria, it could indicate that the improved activity of T5 is due to interactions with the Gram-positive cell wall. It cannot, however, be excluded that T5 is interacting with intracellular bacterial compounds and is only permeable to the Gram-positive cell wall. For thioridazine HCl, it has been proposed that its antimicrobial effect is at least partly due to the compound disturbing the peptidoglycan biosynthesis at a stage that precedes transpeptidation by penicillin-binding proteins [23,24]. This supports observation of the re-sensitizing properties of thioridazine when applied to oxacillin-resistant bacteria. Since T5 does not act synergistically with oxacillin, it most likely has a different mode of antimicrobial action than thioridazine HCl. It could be speculated whether the thioridazine part of T5 is still able to interfere with membrane-related processes, whereas the positively charged sidechain (Scheme 1) would add to the complete disintegration of the membrane, thereby causing lysis of the bacterial cell. Furthermore, the strong bactericidal effect of T5 supports the hypothesis of interference with membrane-related process modes of action. Like thioridazine HCl, T5 is bactericidal by the definition that its MIC and MBC values do not differ more than four-fold [25] (Table 1); furthermore, 4 × MIC is sufficient to eliminate 10^6^ CFU in less than 8 h (Figure 3). In addition, phenothiazines, including thioridazine, have been reported to act as efflux pump inhibiters [9,26]. Since efflux pumps are essential in biofilm formation [26,27], it should be further investigated whether T5 interacts with efflux pumps and possesses biofilm inhibitory properties.

For an antimicrobial intended to treat sepsis, it is critical that an appropriate plasma concentration of the compound can be achieved. The plasma concentration of a compound depends on various factors, such as elimination rate and plasma protein binding (PPB) versus free fraction of the compound [28]. In general, PPB reduces the free fraction of a drug available for bacterial interaction, since only the unbound antibiotic is pharmacologically active. For T5, a high degree of plasma protein binding (90%) was predicted in silico (Table 2). This predication was supported by a significant increase in MIC when 20% human serum was included in the MIC assay (Table 1). High PPB of an antimicrobial does not exclude it from having therapeutic potential, as e.g., ceftriaxone (PPB: 90–95%) and fusidic acid (PPB: approximately 98%) that exhibit high PPB values, yet are highly appreciated antibiotics [28,29]. In such cases, the pharmacokinetics and pharmacodynamics of the compound have to be carefully considered [29]. In contrast to T5, thioridazine was predicted to exhibit a much weaker affinity for plasma proteins (Table 2). This means that a higher fraction of thioridazine molecules are free to cross the BBB and affect the CNS, which could be one of the reasons why severe side effects have been reported previously in phenothiazine-treated animals [10,11]. The PreADMET web application predicts a medium permeability of T5 in Caco-2 cells, and the compound is thus predicted to be well-absorbed after oral administration. This finding must, however, be interpreted with caution. T5 is a constantly charged molecule, and this feature is often unfavorable for passing biological cell linings, which is confirmed by the lack of passage through monolayers of IPEC-J2 hMDR1 cells (Figure 1). Therefore, it is likely that passage over the intestinal lining will be lower than predicted, and in silico and in vitro studies must be applied to confirm whether T5 can actually pass the epithelium lining of the intestine [30].

For non-septic applications, e.g., the topical treatment of infected wounds, PPB would not be relevant. Here, it is of greater importance that the compound is not skin-irritating, and does not penetrate the vascularized dermis of the skin, with a risk of systemic exposure spread rather than having a local effect. Based on the in silico prediction of skin permeability (Table 2), T5 could represent a potential candidate for a topical antimicrobial treatment.

For any medical compound, an assessment of the toxicological profile is essential. Mutagenicity and carcinogenicity were both predicated to be negative for T5. However, medium risk for human ether-a-go-go-related gene (hERG) potassium channel inhibition was predicted. hERG plays an important role in the repolarization of cardiac action potential. It is a serious challenge for a medical compound to reach the market if it has been documented that the compound mediates cardiotoxicity [31]. In fact, cardiotoxicity was one of the primary reasons why thioridazine HCl was withdrawn from the market [29]. A recent study, however, has shown that it is only the (+) enantiomer of thioridazine that processes its cardiotoxic properties, whereas the (+) and (−) enantiomers of thioridazine possess similar antimicrobial activities [32]. Hence, if further in vitro and in vivo studies may confirm the cardiotoxicity of T5, the toxicity could be considerably reduced using only the (−) enantiomer of thioridazine HCl for synthesis of T5.

## 4. Materials and Methods

### 4.1. Synthesis of T5 (1-methyl-2-(2-(2-(methylthio)-10H-phenothiazin-10-yl)ethyl)-1-pentylpiperidin-1-umbromide)

The present design was chosen in order to avoid transport over the–brain barrier by converting thioridazine HCl into a permanently charged derivative. A stirred mixture of thioridazine (0.62 g; 1.67 mmol), 1-bromopentane (2.0 mL; 16 mmol), and acetonitrile (18 mL) was refluxed overnight under an N_2_ atmosphere. After cooling to room temperature, the mixture was added with stirring to diethyl ether (200 mL), causing the product to precipitate. The product was isolated by filtration and dried in a vacuum. The yield was 0.44 g (51%). NMR spectra were recorded on a Bruker 500 MHz instrument equipped with a cryoprobe. HRMS was recorded on a Bruker Solarix XR ESI-MALDI FT-MS. All chemicals were obtained from commercial suppliers, and were used as received.

### 4.2. Transport Assay

At present, a well-characterized cell model resembling the human blood–brain barrier does not currently exist. However, according Di et al. [33], in vitro cellular permeability data, combined with estimates of whether drug compounds are recognized and transported by P-glycoprotein, are predictive for estimating in vivo central nervous system exposure. Therefore, to investigate if the novel thioridazine derivative would have lower BBB permeability compared to thioridazine HCl, the apical-to-basolateral transport of the two different compounds across monolayers of IPEC-J2 MDR1 cells, seeded and cultured on Transwell supports, was measured. The IPEC-J2 MDR1 cells formed electrically tight monolayers and had a high expression of the human efflux transporter P-glycoprotein [34]. Due to the tight barrier and over-expression of human P-glycoprotein, transport experiments across monolayers of the IPEC-J2 hMDR1 cells therefore provided cellular permeability data combined with P-glycoprotein activity.

For transport experiments, both compounds were dissolved in ultrapure water to a concentration of 1 mM and further diluted in Hank’s balanced salt solution (HBSS) (Life technologies, Taastrup, Denmark), supplemented with 0.05% bovine serum albumin (BSA) (Sigma-Aldrich, Brøndby, Denmark) and buffered with 10 mM HEPES (2-[4-(2-hydroxyethyl) piperazin-1-yl] ethanesulfonic acid; AppliChem GmbH, Darmstadt, Germany) to a pH value of 7.4. IPEC-J2 cells were seeded onto permeable supports (T12, Corning cci-3401) and cultured for 15–17 days. The culture medium was changed every other day in both the apical and basolateral chamber.

On the day of transport, the cells were equilibrated to ambient temperatures, and the transepithelial electrical resistance (TEER) was measured across the cell monolayers. Subsequently, the cell layers were washed twice with supplemented HBSS, and the TEER was measured again. The transport experiment was started by replacing the supplemented HBSS buffer in the apical chamber with HBSS solutions of the experimental compounds. Over a period of two hours, samples of 100 µL were taken from the basolateral chamber at *t* = 15, 30, 45, 60, 90, and 120 min. All samples were stored at −20 °C until analysis by means of HPLC–MS. The transport experiment was tested in triplicate. After the transport study was stopped, the cell layers were washed twice with HBSS, and the TEER was measured.

To assess possible alterations of the barrier properties of the IPEC-J2 MDR1 cell monolayers, in a parallel transport experiment, ^14^C-mannitol (0.5 µCi/mL) was added to the two compound solutions. The experiment was performed as described above, with the exceptions that the two compounds were tested in duplicate, and that samples were mixed with 2 mL of Ultima Gold Scintillation Fluid (PerkinElmer, Boston, MA, United States) and analyzed by liquid scintillation counting.

### 4.3. Lactate Dehydrogenase Colorimetric Assay

To investigate if T5 induced cytotoxicity in IPEC-J2 MDR1 cells, LDH release from the cell monolayers was measured in samples of the donor solutions, taken from the apical compartment after 120 min of exposure in IPEC-J2 MDR1 cell monolayers. The cell lysate of cells treated with ultrapure water was included in the analysis as a positive control. Samples were analyzed in duplicate according to manufacturer’s protocol (Roche Diagnostics GmbH, Mannheim, Germany). After incubations with donor solutions (thioridazine HCl or T5) on IPEC-J2 MDR1 cells, absorbance at 492 nm was measured as a mean for LDH release. Hank’s balanced salt solution (HBSS) was used as negative control, and cell lysate from IPEC-J2 MDR1 cell monolayers treated with ultrapure water was used as a positive control.

### 4.4. Antimicrobial Activity

The minimal inhibitory concentration (MIC) and minimal bactericidal concentration (MBC) were determined for eight bacterial strains of human and veterinary clinical importance (Table 1). All strains were stored at −80 °C in a mixture of one part glycerol (50%) and three parts Brain Heart Infusion media (Sigma, Copenhagen, Denmark) until use. The strains were plated on agar plates (Oxoid blood agar base; Oxoid, Roskilde, Denmark) supplemented with 5% bovine blood, and incubated in an appropriate atmosphere (either aerobically or anaerobic). MIC was determined based on CLSI’s guidelines. Briefly, the broth microdilution method was used to determine the MIC. To obtain the desired concentration range (0–256 µg/mL), serial two-fold dilutions of T5 in Müller–Hinton (MH) broth (Sigma-Aldrich, Brøndby, Denmark) were prepared. For each strain, single colonies grown for approximately 24 h on a cultured agar plate were picked and suspended in 0.9% NaCl to reach an optical density equal to that of the 0.5 McFarland standard, using a Sensititre nephelometer (Thermo Scientific, Roskilde, Denmark). The suspensions were then 100-fold diluted in MH broth, and 100 μL of bacterial suspension in MH broth were then added individually into each well of a sterile 96-well plate containing either 100 μL of T5 or the control (pure MH broth), reaching a final inoculum of 5 × 10^5^ colony forming units (CFU). The positive control wells included only MH broth and the bacterial suspension, whereas the negative control wells included only MH broth and T5. The plates were incubated at 37 °C for approximately 24 h. MIC tests were performed in triplicates on separate days. The MIC was defined as the lowest drug concentration that completely inhibited visible bacterial growth. To establish MBC, 100 μL from each well not containing visual growth were plated on blood agar plates and incubated at 37 °C for approximately 24 h. For each strain, MBC was determined as the lowest concentration of T5 that had reduced the CFU of the inoculum (5 × 10^5^ CFU/well) by ≥ 99%. To assess the impact of serum proteins on the activity of T5, the microdilution assays were repeated as stated above, but replacing 40 μL of the MH broth with 40 µL human serum (Sigma-Aldrich, Brøndby, Denmark), resulting in a 20% serum concentration in the assay.

### 4.5. Growth and Viability Assays

Viability assays of *S. aureus* JE2 in the presence of 0, 1, 2, 4, and 8 µg/mL T5 in MH broth in eight-hour intervals was performed. MH cultures of *S. aureus* JE2 in early exponential phase were transferred to 100 mL flasks, and the various concentrations of T5 was added to each flask. The flasks were placed in a 37 °C water bath with 100 RPM shaking. The number of CFU for each T5 exposure condition was determined at seven time points during the eight hours. For each time point, 100 µL culture from each flask was submitted to serial 10-fold dilution in a 0.9% NaCl solution, and 100 µL from each dilution was plated on an MH agar plate. The plates were incubated for 24 h at 37 °C, and CFU were determined by counting colonies on the plates. The experiment was repeated in biological triplicates.

### 4.6. Oxacillin Synergy Assessment

To investigate if T5 is interacting synergistically with oxacillin, a checkerboard assay was performed. In addition, a checkerboard assay of thioridazine HCl combined with oxacillin was performed as a control. For each checkerboard assay, a total of 50 μL of MH broth was distributed into each well of the microdilution plates. A T5 or thioridazine HCl solution was serially diluted along the ordinate, while the oxacillin was diluted along the abscissa. An inoculum equal to a 0.5 McFarland turbidity standard was prepared for the MIC/MBC assays. Each microtiter well was inoculated with 100 μL of a bacterial inoculum at 5 × 10^5^ CFU/mL, and the plates were incubated at 37 °C for 24 h under aerobic conditions, with a final volume of 200 µL. After incubation, a fractioned inhibitory index (ΣFIC) was calculated for each well without visual recognizable growth. The ΣFICs were calculated as follows: ΣFIC = FIC A + FIC B, where FIC A is the MIC of thioridazine HCl or T5 in the combination, or the MIC of thioridazine HCl or T5 alone. FIC B is the MIC of oxacillin in the combination or the MIC of oxacillin alone. The combination is considered synergistic when the ΣFIC is ≤ 0.5, indifferent when the ΣFIC is > 0.5 and < 2, and antagonistic when the ΣFIC is ≥ 2.

### 4.7. In Silico Analysis of Pharmacokinetics and Toxicity

The web-based application PreADMET [35] was used for in silico predicting of T5 characteristics, including human intestinal absorption, plasma protein binding, caco-2 cell permeability, skin permeability, mutagenicity, and carcinogenicity. For data input for the PreADMET application, the SMILES formula of T5 ([Br-].CCCCC[N+]1(C)CCCCC1CCN1C2=CC=CC=C2SC2=CC=C(SC)C=C12) and thioridazine HCl (C1=4(C(SC=3(C(N1CCC2([N+](CCCC2)C))=CC=CC=3))=CC=C(C=4)SC).[Cl-]) were converted to MOLfiles using Cheminfo.org.

## 5. Conclusions

Here we have synthesized a thioridazine derivative that exhibits low blood–brain barrier permeability and highly improved antimicrobial activity, compared to its “mother” compound. In vitro investigations and in silico predications indicated that T5 could represent a novel antimicrobial compound for the clinical treatment of infections caused by Gram-positive bacteria. These data strongly call for further pharmacokinetic and pharmacodynamic studies to fully investigate the therapeutic potential of T5, as well as a full elucidation of the antimicrobial mode of action. Furthermore, this study demonstrates that modest chemical modification may induce a compound to change from an antipsychotic to an antibiotic. This perhaps opens a new door in the search for novel antimicrobials that can minimize the global issue regarding emerging multidrug-resistant pathogens.

## Figures and Tables

**Table 1:** Antimicrobial activity of T5.

Species	Strain	Origin	MIC (µg/mL)		MBC (µg/mL)
			MH Broth	MH Broth + 20% Human Serum	
*Staphylococcus aureus*	JE2 (USA300)	Human clinical isolate	2	16	2
*Staphylococcus aureus*	ATCC BAA-1556	Human clinical isolate	2	32	2
*Staphylococcus aureus*	CC398	Veterinary clinical isolate	1	16	1
*Enterococcus faecalis*	72B6	Veterinary clinical isolate	4	ND	4
*Enterococcus faecium*	ATCC 700221	Human clinical isolate	8	ND	8
*Proteus vulgaris*	4663	Veterinary clinical isolate	64	ND	64
*Escherichia coli*	E2	Human clinical isolate	64	ND	64
*Escherichia coli*	APEC O2	Veterinary clinical isolate	32	ND	32

The table shows the minimal inhibitory concentration (MIC) with and without serum-added Müller–Hinton (MH) broth and minimal bacterial concentration (MBC) for T5. Five Gram-positive and three Gram-negative clinical isolates were tested. ND: not determined.

**Table 2:** In silico predictions of T5 and thioridazine HCl.

Feature	T5	Thioridazine HCl	Range
Plasma protein binding (%)	90.9	62.5	>90%: chemicals strongly bound
Blood brain barrier penetration (*C_brain_*/*C_blood_*)	0.1	0.5	2.0–0.1: middle absorption to CNS
Skin permeability (logKp, cm/hour)	−1.92	−3.7	Not defined
Caco-2 cell permeability	21.78	31.9	4–70: middle permeability
Human intestinal absorption (%)	98.26	94.4	70–100%: well-absorbed compounds
Ames test (TA100)	Negative	Positive	-
Carcinogenicity	Negative	Positive	-
hERG inhibition	Medium risk	High risk	-

The table shows important pharmacokinetic and pharmacodynamic parameters for T5 and thioridazine HCl predicted by the web-based application PreADMET.
